# Supplementary material for: Role of ambient pressure in self-heating torrefaction of dairy cattle manure
Source: PLoS One. 2020 May 29;15(5):e0233027. doi: 10.1371/journal.pone.0233027 (PMC7259747; doi:10.1371/journal.pone.0233027)
Supplement: Attachment — Submitted filename: ResponsetoReviewers.docx [file pone.0233027.s001.docx]

**Response to Reviewers**

We thank the academic editor and the reviewer for their thoughtful and supportive comments. We have revised our manuscript in response to their suggestions and hope that this improved manuscript is acceptable for publication in *PLOS ONE*.

**Comment from Academic Editor:**

*The manuscript is missing critical important information which must be added before the manuscript can be considered for publication. The use of replicate for the data presented as well as how the data was statistically analyzed must be present. Otherwise the manuscript is written in very good English and it is very easy to follow and understand. The only drawback is the methodology section missing the above mentioned info.*

**Response:**

We reperformed experiments in the revised version of the manuscript to ensure reproducibility. The average values ± standard deviations of two replicates are reported. The obtained data were subjected to a two-way ANOVA followed by Tukey’s test. All data on the properties of biochar have been updated, but these changes did not affect the interpretation of the results and conclusions. We added this information as well as an improved explanation of the experimental design in the Materials and methods section.

**Comment from Reviewer #1:**

*Principal Comments: Material and Methods section is short and lacking sufficient information. Authors are strongly advised to include name of all standard methods (ASTM- American Society for Testing and Materials) followed to perform ultimate and proximate analyses along with a very brief description of the measurement methods and instruments used. Example are as follows: Standard Methods Instruments/ Model Measurement methods*

*Proximate*

*Volatile Matter ASTM D7582 Leco TGA 701 Thermogravimetric Analyzer Mass loss (% dry basis) of the specimen when heated at 950ºC in presence of oxidizing gas for 2 h.*

*Heating Value*

*(Calorific Value) ASTM D5865 Parr 6200 Calorimeter, Parr Instruments (Moline, IL, USA) Oxygen bomb calorimeter*

**Response:**

We appreciate your advice. Although we added the instrument for VM determination along with a description of the measurement method in Sample Analyses, we cannot show the names of all standard methods for proximate, ultimate, and calorific analyses because the analyses were not strictly based on standard methods. However, as all the analyses were performed by methods similar to the standard method (ASTM E871-82, ASTM E1755-01, and ASTM D2015 for moisture, ash, and calorific value), we believe that the data obtained are reliable.

*Statistical analysis: A small section of statistical methods and analysis should be included. Nothing is mentioned about replications whether samples were replicated or duplication or subsamples. To publish this article, sample should be duplicated. This is major drawback of this article.*

**Response:**

We added explanations of the statistical analyses as well as the experimental design in the Materials and methods section.

**We look forward to hearing from you regarding our submission. We would be pleased to respond to any further questions and comments that you may have.**
